# Cellular imaging of inherited retinal diseases using adaptive optics

**DOI:** 10.1038/s41433-019-0474-3

**Published:** 2019-06-04

**Authors:** Jasdeep S. Gill, Mariya Moosajee, Adam M. Dubis

**Affiliations:** 1 UCL Institute of Ophthalmology, 11-43 Bath Street, London, EC1V 9EL UK; 2 NIHR Biomedical Research Centre at Moorfields Eye Hospital NHS Trust and UCL Institute of Ophthalmology, 162 City Road, London, EC1V 9PD UK; 3 Great Ormond Street Hospital for Children, Great Ormond Street, London, WC1N 3JH UK

**Keywords:** Prognostic markers, Predictive markers

## Abstract

Adaptive optics (AO) is an insightful tool that has been increasingly applied to existing imaging systems for viewing the retina at a cellular level. By correcting for individual optical aberrations, AO offers an improvement in transverse resolution from 10–15 μm to ~2 μm, enabling assessment of individual retinal cell types. One of the settings in which its utility has been recognised is that of the inherited retinal diseases (IRDs), the genetic and clinical heterogeneity of which warrants better cellular characterisation. In this review, we provide a summary of the basic principles of AO, its integration into multiple retinal imaging modalities and its clinical applications, focusing primarily on IRDs. Furthermore, we present a comprehensive summary of AO-based cellular findings in IRDs according to their associated disease-causing genes.

## Introduction

Since its first use in retinal imaging just over 20 years ago [1], adaptive optics (AO) has undergone immense growth in its applications amongst vision scientists and clinicians. This technique has allowed imaging of the living retina at a cellular resolution, leading to revolutionary changes in our understanding of retinal diseases and especially those of monogenic aetiology. In doing so, it has opened exciting avenues for research, monitoring retinal diseases [2–4], and providing new tools for refining diagnosis [5, 6].

Adaptive optics was originally a component added to astronomical telescopes to rectify loss of resolution from atmospheric irregularities (wind and moisture) [7]. Its uses have since evolved and have been employed in the fields of microscopy, communication and medicine. In ophthalmic applications, AO is a tool by which monochromatic aberrations present in the optical path of the eye are measured and then compensated. In reference to the eye, the measurement and full wavefront correction can improve the transverse optical resolution from 10–15 μm to ~2 μm. This enhanced resolution enables visualisation of numerous retinal cell types including ganglion cells [8, 9], photoreceptors [10–13], and retinal pigment epithelial (RPE) cells [14, 15]. AO, as a bolt-on technique, has been integrated into multiple existing retinal imaging systems, including flood illumination ophthalmoscopy (FIO) [1, 16], scanning laser ophthalmoscopy (SLO) [17, 18] and, most recently, optical coherence tomography (OCT) [19, 20]. Although it is yet to be in widespread clinical use, the advent of AO has truly redefined the possibilities for in vivo retinal imaging and transformed the ophthalmic research space.

One of the most common targets of ophthalmic AO imaging has been in patients with mendelian inherited retinal diseases (IRDs). Patients with IRDs are clinically and genetically heterogeneous; over 250 disease-associated genes have been identified to date, with patients displaying retinal dysfunction, which can be either stationary or progressive, leading to sight loss [21]. Given that the primary cell types affected in IRD patients are photoreceptors and RPE cells, AO-based retinal imaging provides a vital tool for shedding light on cellular pathogenesis, disease progression and its clinical correlation. This is especially true as studies show the detection of microstructural retinal changes prior to functional changes occurring in a patient’s vision [22, 23]. Similarly, AO plays a crucial role in sensitively assessing the efficacy of interventions rapidly developing for IRDs, including stem cell, gene replacement and gene modification therapy [24]. In view of the significance of AO retinal imaging in IRDs, our review summarises the cellular changes corresponding with mutation-specific disease, whilst exploring its basic principles, uses and clinical applications.

## Basic principles of adaptive optics

Optical aberrations are deviations of incoming light rays from the ideal path, such that they do not converge into a single point of focus on the retina. Aberrations can largely be attributed to the three optical elements of the eye: the cornea, the pupil and the crystalline lens, however other components (aqueous/vitreous humour and retinal tissue) can also contribute [25]. Whilst the corneal surface contributes to approximately two-thirds of the eye’s optical aberrations, the crystalline lens is largely responsible for the remainder through its shape, thickness and cellular alignment. Sitting in-between the cornea and lens, the pupil also affects optical quality by regulating the quantity of light entering and exiting the eye during imaging [26]. Optical aberrations can be classified as being of either low- or high-order. Low-order aberrations, such as defocus and astigmatism, are of greatest magnitude, but corrected with glasses or contact lenses. In contrast, high-order aberrations, such as keratoconus, constitute a small proportion of optical aberrations and have proven more challenging to correct for. Although the effects of many optical aberrations are only detectable with specialist testing [27], others can significantly distort both a patient’s vision and their retinal images.

**Fig. 1:**
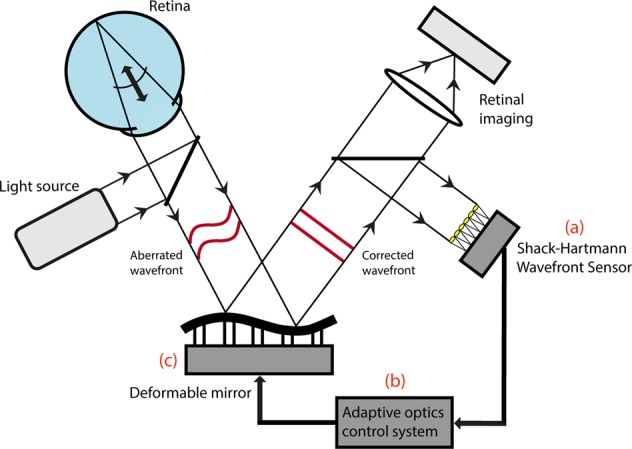
Schematic diagram of an adaptive optics-assisted retinal imaging system. An AO system measures the aberrated wavefront using a wavefront sensor (**a**), and compensates for this using a wavefront corrector (**c**). These usually take the form of a Shack-Hartmann Wavefront Sensor (SHWS) and deformable mirror, respectively. These two components are connected by a central control system (**b**). The resulting AO-corrected retinal image is recorded using a high-resolution camera

AO-aided imaging compensates for a patient’s individual optical aberrations to provide a high-resolution view of the retina. These systems classically consist of three major components: a wavefront sensor, a wavefront corrector, and a control system linking the prior two constituents (Fig. 1). The wavefront sensor measures the patient’s ocular aberrations, whilst the control system interprets these to communicate the required adjustments to the wavefront corrector. In the closed-loop configuration conventionally used, the wavefront sensor is positioned after the wavefront corrector to provide feedback regarding residual aberrations until the wavefront matrix is minimised. This is ideally the diffraction limit of the patient’s eye, however this is not always possible [28].

### Wavefront sensor

The Shack-Hartmann Wavefront Sensor (SHWS) is the most commonly used wavefront measurement technique in ophthalmology. It consists of an array of micro-lenslets located in front of an area detector [29]. In the SHWS, the aberrated light leaving the eye illuminates micro-lenslets to produce an arrangement of spots on the detector. Since each micro-lenslet corresponds to a location at the pupil, the SHWS assesses how much each detected spot position deviates from its intended position. The calculated deviation is used to make inferences about the wavefront slope and amplitude at each location and is compiled across the pupil, thus determining the type and magnitude of optical aberration across the wavefront.

### Wavefront corrector

Numerous techniques are available to correct the wavefront and are generally divided into two categories: piston-segmented devices and continuous surface mirrors. The most commonly used wavefront corrector, the deformable mirror, is comprised of a reflective faceplate deflected by a series of actuators, which can be either segmented or continuous. As the segmented deformable mirror leads to more diffraction-induced spurious effects, continuous deformable mirrors are the mainstay of use in AO retinal imaging. Over the last decade, a dual deformable mirror configuration, known as a ‘woofer-tweeter system’, has come into use in which the differing specifications of each mirror confers correction of a wider range of aberrations [30–33]. The woofer-tweeter arrangement involves both deformable mirrors conjugated to the plane of the pupil. In contrast, multiple groups have applied a dual-conjugate configuration in which one deformable mirror conjugates to the pupil whilst the other deformable mirror conjugates to a plane in front of the retina [34, 35]. This enables aberration correction for a greater field of view, allowing wide-field high-resolution retinal imaging.

### Wavefront sensor-less systems

More recently, a wavefront sensor-less AO correction system has been implemented in ophthalmic imaging, using image quality in place of a wavefront sensor to correct for aberrations [36–38]. Whilst this offers simplification of current AO systems, the serial assessment required of parameters, such as image sharpness, denotes an increased aberration correction time. This trade-off has implications in patients with temporally fluctuating aberrations or eye movements (nystagmus). Efforts are thus being made to reduce the time required for wavefront sensor-less correction to allow its application to become more widespread. This reduction is being spurred by the continual increase of computing power (CPU cores) and newer array-based computing possibilities (GPU) [39].

## Retinal imaging modalities using adaptive optics

### Adaptive optics flood illumination ophthalmoscopy

The first published AO retinal images were produced at the University of Rochester in 1997, where this technique was incorporated in FIO [1]. Its original applications were to understand cone density and arrangement underlying psychophysical responses. It was several years before AO use in retinal imaging progressed from understanding normal vision, to investigating retinal pathology [40]. Further complicating the uptake of AO-based imaging in ophthalmic practice was the reliance on custom-built systems, oversized for the clinical setting. However, the first compact AO retinal imaging system became commercially available in 2011 in the form of the rtx1 (Imagine Eyes, France), providing a 4 × 4˚ (~1.3 × ~1.3 mm) retinal view [41].

The advantages of AO-FIO include its incorporation of a spinning diffuser to eliminate speckle from its light source. Conversely, it is limited by a poor axial resolution (~300 μm), which reduces cone photoreceptor contrast and decreases repeatability of longitudinal cone density measurements [42, 43]. The efficiency of AO-FIO was previously restricted by its long imaging time per frame due to light source and detector technologies. However, the advent of the superluminescent diode (SLD) as a light source, paired with a high-speed charge-coupled device, has enabled faster frame rates [16]. This has conferred AO-FIO with the capacity to record real-time retinal videos, particularly in the context of retinal perfusion [44]. In one of the earliest studies of its use in IRDs, Choi et al. [45] employed AO-FIO to directly correlate the extent of cone abnormalities with functional loss of vision in patients with retinal dystrophy.

### Adaptive optics scanning laser ophthalmoscopy

The next AO ophthalmic imaging breakthrough arrived in the form of the AOSLO in 2002 [18], which improved upon the AO-FIO’s image contrast and added the ability to optically section. In AOSLO, a single-point light beam is raster scanned across the retina, with scattered light from each point recorded by a light-sensitive detector to construct an image. This system allows the incorporation of multiple detection modes, including confocal, offset pinhole and non-confocal methods. In confocal imaging, a pinhole is positioned in front of the detector, which allows the rejection of light scattered from areas other than those within the retinal focal plane. As a result, the image at the point of focus is of significantly greater contrast and allows a system to be designed that enables retinal sectioning. Confocal AOSLO is particularly useful in permitting the imaging of rod photoreceptors [10, 46, 47] and elements of the optic nerve, including the nerve fibre layer [48] and lamina cribrosa [49].

**Fig. 2:**
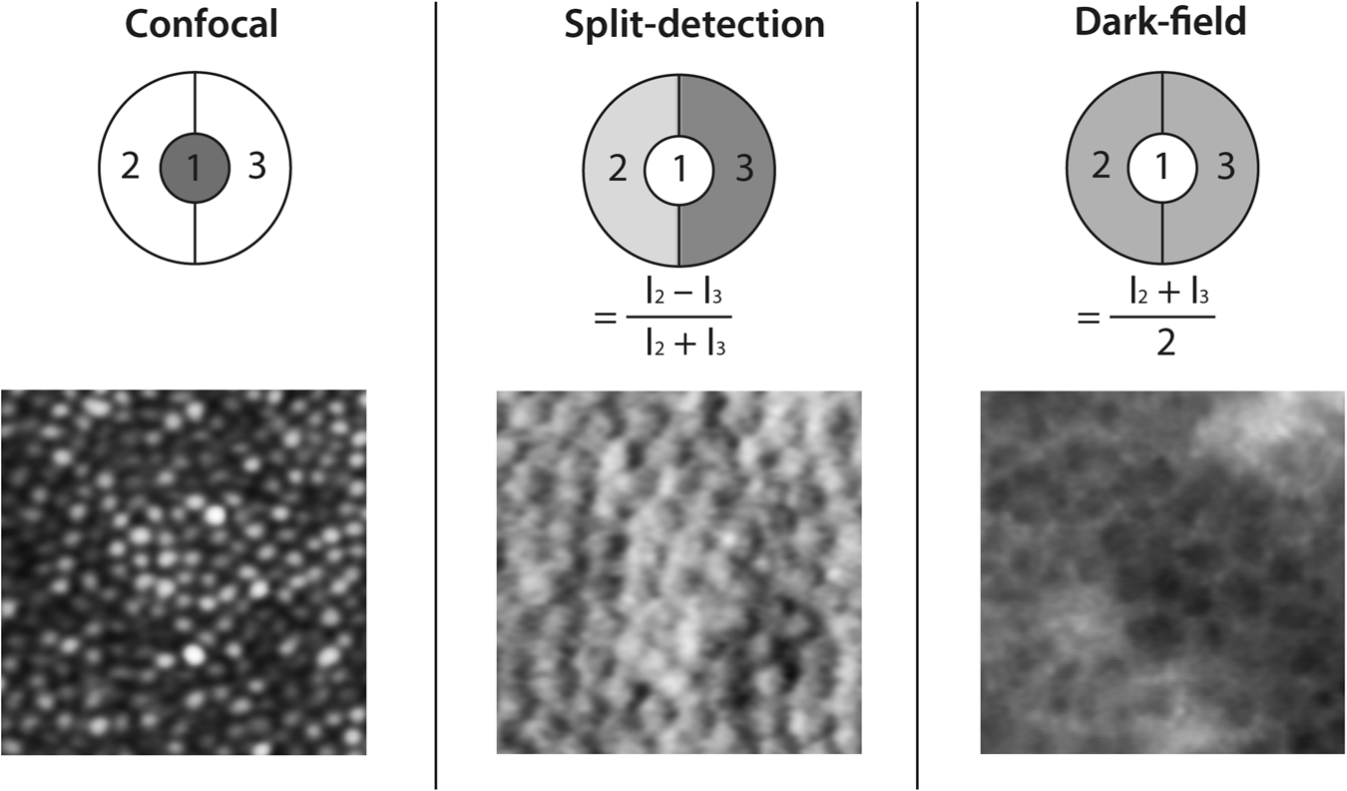
Comparison of detection modes using adaptive optics scanning laser ophthalmoscopy (AOSLO) in healthy retina. Detector views of the annular reflective mirror (top) represent the relative contributions of scattered/non-scattered light in producing each AOSLO modality image (bottom). Confocal imaging utilises light within the retinal focal plane, whereas non-confocal (split-detection and dark-field) images are produced from scattered light. Individual photoreceptor cells can be identified on both confocal and split-detection imaging. Dark-field imaging reveals the underlying retinal pigment epithelium (RPE) as a hexagonal mosaic. The AOSLO images shown are from different retinal locations in the same healthy eye

**Fig. 3:**
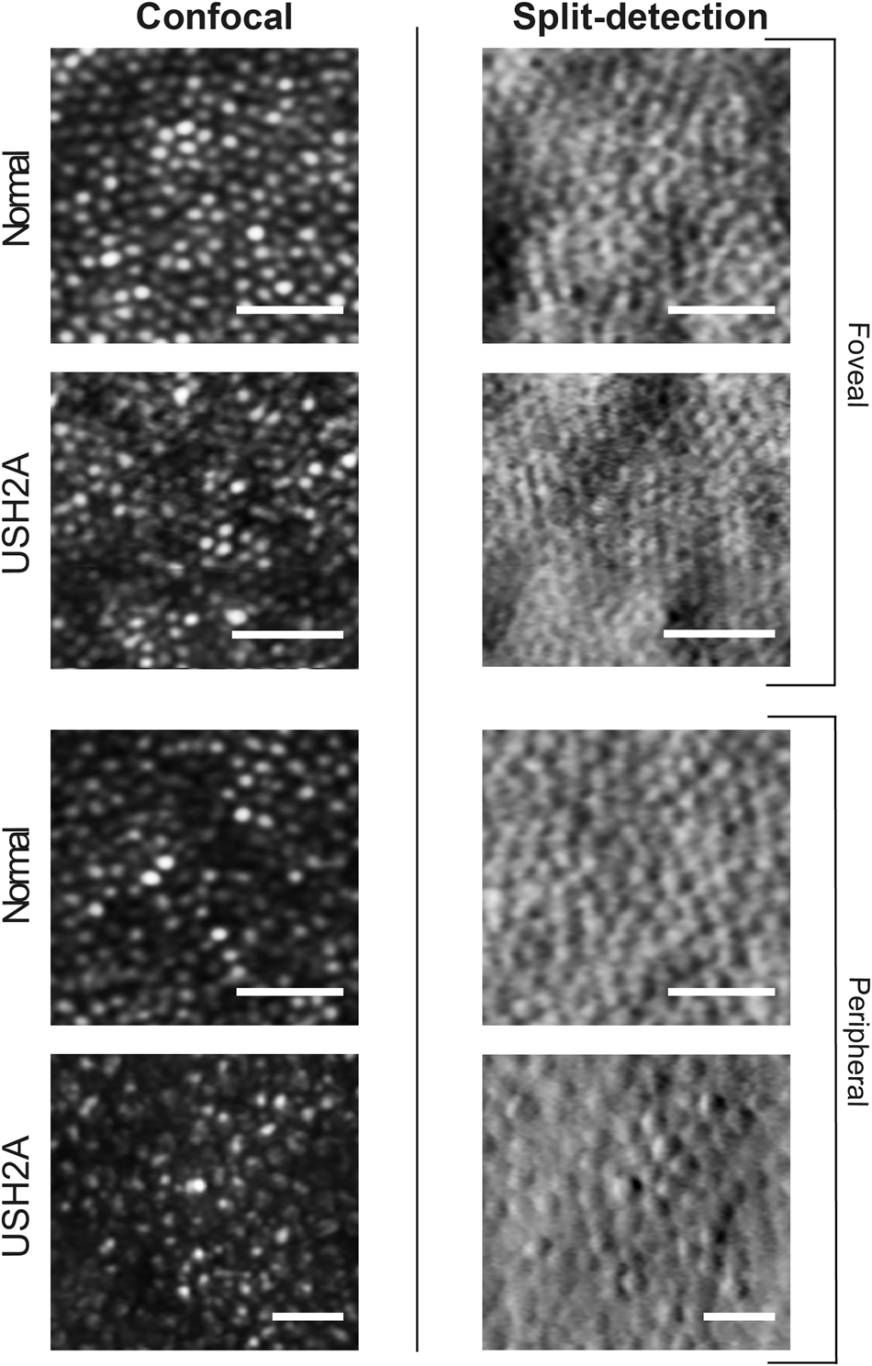
Comparison of confocal and non-confocal (split-detection) AOSLO in a healthy control and an *USH2A-*associated type II Usher syndrome patient. AOSLO imaging of a 35-year-old male healthy control shows a regular and densely packed photoreceptor mosaic at the fovea (top left), with larger and less dense photoreceptors in the periphery. Although these photoreceptors demonstrate varying degrees of reflectance, there are no prominent dark regions. In contrast, imaging of a 43-year-old female with *USH2A*-associated retinitis pigmentosa reveals a disrupted foveal mosaic with non-waveguiding areas, which increases in severity peripherally. Split-detection imaging (bottom right) of non-waveguiding areas in the peripheral confocal mosaic (bottom left) shows remnant cone inner segments in this patient. Scale bar = 10 μm

Over the last 5 years, the advantages of non-confocal AOSLO have also emerged. In contrast to confocal AOSLO, this involves collecting scattered light surrounding the point of focus instead of the confocal signal itself. The resultant annular signal enables effective visualisation of the RPE without the need for autofluorescence, known as ‘dark-field imaging’ [15]. Alternatively, this signal can be divided in two to reveal inner segments of photoreceptors not seen on confocal AOSLO, termed ‘split-detection imaging’ (Fig. 2) [50]. This confers advantages in IRDs in which photoreceptor outer segment (OS) composition and alignment are affected, such as achromatopsia [51], choroideremia [52], Stargardt disease [53], and Leber congenital amaurosis [54]. There is, therefore, strong rationale for the complementary use of both confocal and non-confocal AOSLO in the assessment of retinal structure, especially in IRDs (Fig. 3) [23, 55–57]. Disadvantages of AOSLO include its propensity for image distortion, as well as the inefficiency of its serial image creation [58].

### Adaptive optics optical coherence tomography

The latest advent in retinal imaging has been the incorporation of AO in OCT. Now in widespread clinical use, the introduction of OCT in 1991 made possible a cross-sectional view of the retina previously only seen ex vivo as a histological section [59]. Although native OCT provides a subcellular axial optical resolution, its axial and transverse resolution is still limited by optical aberrations, thus making its partnership with AO ideal. AO-OCT has facilitated three-dimensional visualisation of single photoreceptors [60], retinal nerve fibre bundles [61], the lamina cribrosa [62], and retinal vasculature [63]. An exciting recent development is the simultaneous use of AOSLO and AO-OCT, which offers the ultrahigh axial resolution of the latter whilst utilising eye tracking advantages of the former to reduce the effect of saccade [13, 64].

### The effect of optical resolution and image sampling on cell visibility

One challenge in understanding the AO imaging literature has been discrepancies in interpretations of the same disease, due to differences in capabilities between the various AO-enabled platforms and devices [56, 57, 65–68]. These variations may be initially interpreted as inherent differences in resolution across AO imaging modalities. However, this is more often due to a difference in image sampling, rather than optical resolution. For instance, foveal cones and near-fovea rods (<2 mm from centre) are roughly 2 µm in diameter [69], which is near the theoretical optical resolution for a nominal eye [70, 71]. Rod photoreceptors change in size modestly, reaching roughly 2-4 µm in the peripheral retina (>8 mm from centre). In comparison,  cone photoreceptors  markedly increase in diameter from their 2 µm foveal size, to >10 µm by 3 mm from the centre of fixation [69, 72]. The differences in photoreceptor diameter have contributed towards their variable visibility across modalities. Rod photoreceptors were first observed in IRD patients using FIO technology [73] in 2006, and seen using the same technology in normal subjects in early 2011 [74], before first being published using AOSLO [10] later that year. Subsequently, rods have also been visualised using AO-OCT methods [13, 60]. Many of the early FIO systems had a sampling resolution between 1–2 µm per pixel, including commercially available systems [11]. Alternatively, custom built AOSLO systems have been designed with sub-micron sampling resolutions [71, 75] which, due to Nyquist limits and contrast enhancements from confocal imaging [76], are better positioned to take advantage of and achieve the theoretical limits of resolution. Therefore, the common differences observed between modalities to date has been due to differences in image sampling. The interaction between sampling, optical resolution and device design has been reviewed elsewhere [70, 71].

## Applications of adaptive optics in inherited retinal diseases

The application of AO in IRDs has progressed from exploring disease genotype-phenotype correlations [77–79], to longitudinal assessment of disease progression using cellular metrics as potential trial endpoints [2–4, 80]. To enable the latter, AO-aided imaging has demonstrated the ability to image the same retinal area with microscopic precision longitudinally [81, 82]. Several aspects of retinal microstructure, such as photoreceptor density and morphology, have consequently emerged as significant to the disease process and assisted in characterisation of each condition. These have been proposed as metrics for evaluating therapeutic potential and efficacy in patients [83, 84], and are therefore of great clinical relevance.

To understand the differences between normal and abnormal cell densities, an understanding of normal variability must be assessed. Most optical imaging literature still refer back to the seminal histological work by Curcio et al. [69], to assess the density and organisation of the rod and cone photoreceptors. Several groups have since looked at larger cohorts of healthy retina using AO-aided imaging, and have now developed extensive normal subject photoreceptor density data [10, 82, 85–87]. It is largely agreed that the very centre of the fovea is rod-free, expanding out to a region of 250–500 µm in diameter [69, 88]. Cone photoreceptor density drops precipitously from ~300,000 to 100,000 photoreceptors per mm^2^ as you move from the centre to the edge of this region (~50% less) [69, 72, 82, 88]. The photoreceptor density continues to drop to nominally 5000 mm^2^ by 3 mm from the centre, but this is affected by several factors such as axial length [69, 82, 85]. Over the same area, rod photoreceptors  transition from absence at the centre to a peak of 125,000 rods per mm^2^ at about 4 mm radius, before slowly decreasing in density as you move to the far periphery.

### Photoreceptor-based metrics

**Fig. 4:**
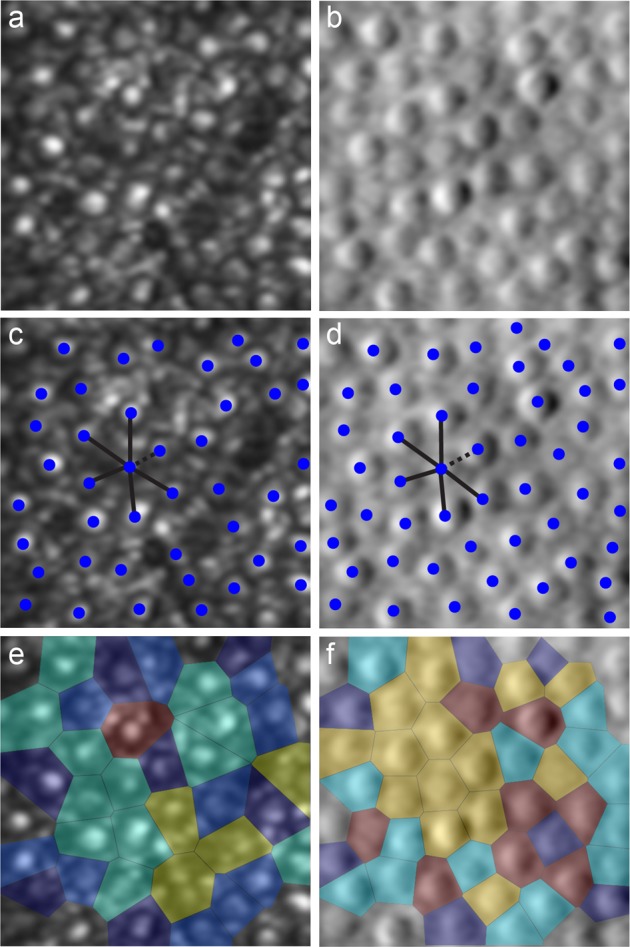
Application of photoreceptor mosaic-based metrics. Shown here are simultaneously acquired confocal (**a**) and split-detection (**b**) AOSLO images. Photoreceptors can be directly identified using automated or manual methods (blue spots) in the confocal (**c**) and split-detection (**d**) image. The modalities provide different rates of identifiable cones. Once cone centres are identified, the nearest neighbour distance (dashed line) or mean inter-cone distance (average of all lines) can be measured. Additional metrics include Voronoi diagrams of the foveal cone photoreceptors (**e**, **f**), the colouration of which is used to illustrate the number of neighbours each cell has surrounding it

The ideal photoreceptor mosaic is organised in a regular triangular lattice with hexagonal packing [89]. Several metrics aim to detect and quantify photoreceptor loss in this arrangement, and can be broadly categorised into measures of density, spacing and regularity (Fig. 4) [90]. Cone density is the most commonly used mosaic metric, with good reliability and repeatability established in healthy controls [91–93]. However, the comparative difficulty of cone identification in retinal degenerations has rendered this metric variable when applied to patients with Stargardt disease [94], *RPGR*-associated retinopathy [94], and achromatopsia [79]. In addition to inter-disease variances, several studies demonstrate inter-imaging modality differences in cone density reliability, with split-detector AOSLO conferring the best results [94, 95]. Limitations in reliability and repeatability are similarly reported for cone spacing metrics in IRDs [96], largely due to differences in mosaic sampling window size and areas with increased cone reflectivity loss. Nevertheless, both cone density and spacing were successfully used by Talcott et al. [97] as measures of treatment efficacy in patients with retinitis pigmentosa (RP) and Usher syndrome, in which ciliary neurotrophic factor administration led to a reduced rate of cone loss.

Photoreceptor reflectance properties have also been explored for their utility as cellular metrics. On viewing the in vivo retina using confocal AOSLO, bright Gaussian profiles representing individual waveguiding photoreceptors are visible, with their intensity exhibiting temporospatial variation [46, 98]. The degree of cone illumination is proposed to reflect OS function [99], and is supported by functional imaging studies demonstrating stimulus-evoked increases in cone brightness [100–102]. In the context of IRDs, Dubis et al. [83] validated cone reflectance as a functional metric in achromatopsia by correlating greater mean cone reflectivity with better residual cone function. In corroboration with this, a hyporeflective annulus surrounding a central region of atrophy was noted in Stargardt disease, which correlated with areas of cone loss [103]. However, occurrence of rod hyperreflectivity in Oguchi disease [104] supports an alternative hypothesis linking photoreceptor intensity to their OS length [105], which are reported as shortened in this condition [106]. Raising further questions regarding the validity of this metric, Bruce et al. [107] reported weakly waveguiding cones in healthy retina which performed normally on functional testing with cone-based microperimetry.

Finally, AO imaging may be used to quantify morphological changes in individual photoreceptors during the degeneration process [108], namely in OS length and inner segment (IS) diameter. Jonnal et al. [109] recently devised a method using phase-sensitive AO-OCT to detect changes in OS length down to 45 nm, whilst Liu et al. [55] have utilised split-detection AOSLO to measure remnant cone IS. Despite its use as a marker by Sun et al. in RP [23] and choroideremia [57], the utility of IS diameter is confounded by enlargement of some cones secondary to neighbouring cone loss rather than their own degeneration [110]. Further studies using morphology-based metrics are required to better elucidate their utility.

### RPE-based metrics

The use of RPE-based metrics for retinal diseases is one of the most recent and exciting research avenues in AO-based imaging. The RPE maintains a close structural and functional relationship with its adjacent photoreceptors, holding essential roles in OS phagocytosis, the visual cycle, light absorption and transepithelial transport [111, 112]. In spite of its dysfunction and/or atrophy underlying multiple IRDs, including RP [113], biomarkers signifying its change have been limited. This is mainly attributable to difficulty in its direct visualisation, first being imaged after the degeneration of its overlying photoreceptors [114]. However, Liu et al. [14] recently applied AO-OCT to provide three-dimensional characterisation of RPE cells, proposing quantification of their organelle motility as a functional metric [115]. Potential for RPE density as a biomarker has also emerged by implementation of AO in infrared autofluorescence imaging (AO-IRAF) [116]. Application of AO-IRAF in an RP patient delivered a resolution that enabled precise calculation of RPE density and RPE-to-photoreceptor ratio.

### Comparison of metrics

Selection of the ‘best’ metric in a patient or clinical study is based on two factors: its functional relevance, and its sensitivity in detecting change. At present, both are only well-characterised in photoreceptor mosaic-based metrics [90]. Morphology- and RPE-based metrics are relatively well understood regarding their structure-function relationships, but warrant further exploration to assess their sensitivity and robustness. Photoreceptor reflectance, on the other hand, remains ambiguous in both its origin and utility. Although based on data from healthy controls, Cooper et al. [90] showed significantly greater sensitivity of mosaic regularity metrics in identifying cone loss than of spacing metrics, suggesting their utility in longitudinal disease monitoring. However, combining multiple metrics, as opposed to using a single measure, may confer the greatest accuracy in monitoring retinal disease [87, 117].

A major issue in the comparison of metrics, and in their robust application, is the lack of multicentre standardisation. This includes the absence of a central normative dataset to assess findings against, as well as variation in the size of retinal sampling windows used. These differences can cause discrepancies in results, and thus prevent us from assimilating data from different research groups when evaluating metric utility.

## Findings using adaptive optics in inherited retinal diseases

**Fig. 5:**
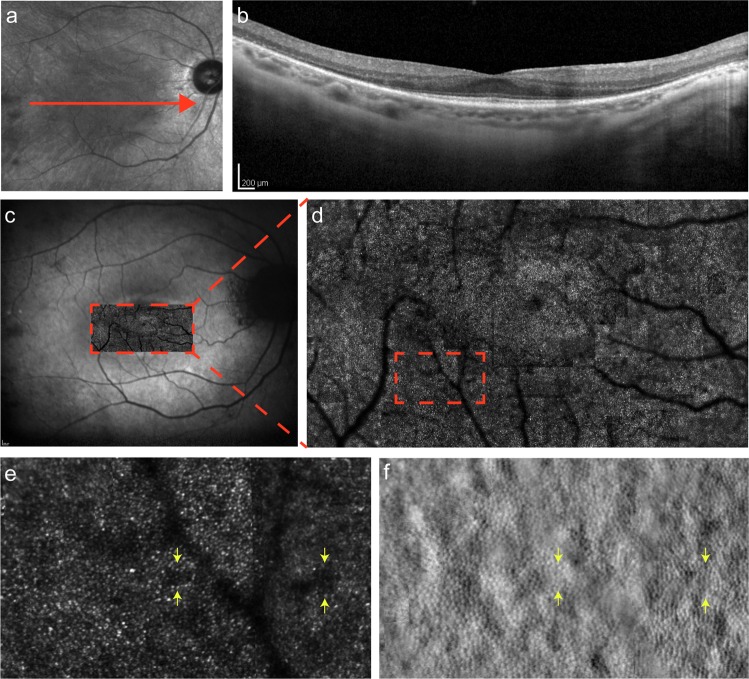
Multimodal imaging of *USH2A*-associated type II Usher syndrome. **(a)** Infrared reflectance (IR) fundus photograph, with the central red arrow representing the optical coherence tomography (OCT) section shown in (**b**). (**c)** Fundus auto-fluorescence (FAF) photograph with confocal adaptive optics scanning laser ophthalmoscopy (AOSLO) imaging of the fovea and its surrounding region superimposed and magnified in (**d**). Further magnification of a peripheral area (dashed rectangle) in the confocal image (**e**), and its corresponding AOSLO split-detection image (**f**), shows intact photoreceptor inner segments in non-waveguiding areas of the confocal mosaic (yellow arrows)

**Fig. 6:**
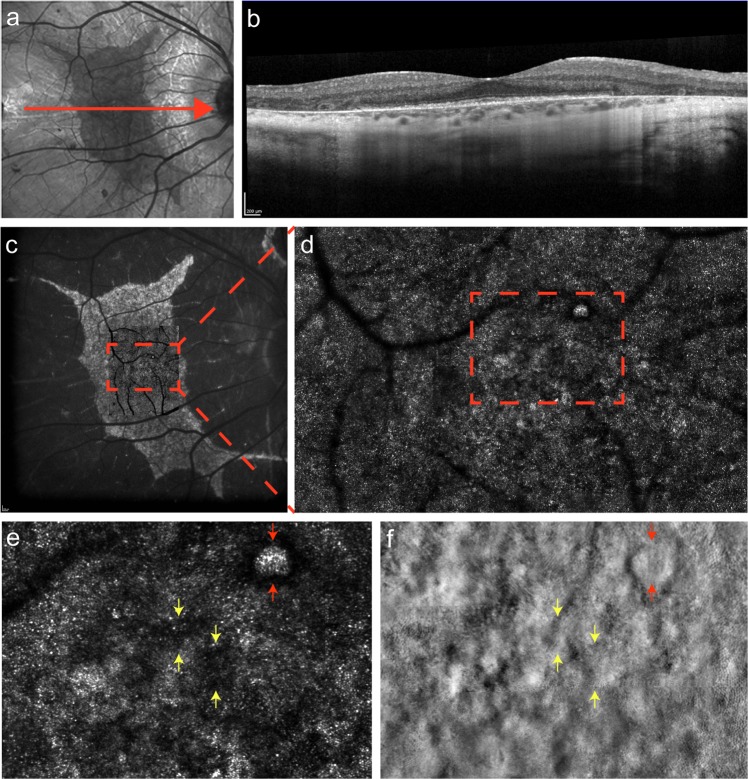
Multimodal imaging of *CHM-*associated choroideremia. **(a)** IR fundus photograph, with the central red arrow representing the OCT section shown in (**b**). **(c)** FAF photograph with confocal AOSLO imaging of the fovea and its surrounding region superimposed and magnified in (**d**). Further magnification of a perifoveal area (dashed rectangle) in the confocal image (**e**), and its corresponding AOSLO split-detection image (**f**), shows a well-circumscribed island of intact photoreceptors surrounded by non-waveguiding cones (red arrows). Intact photoreceptor inner segments are seen in non-waveguiding areas of the confocal mosaic (yellow arrows)

**Fig. 7:**
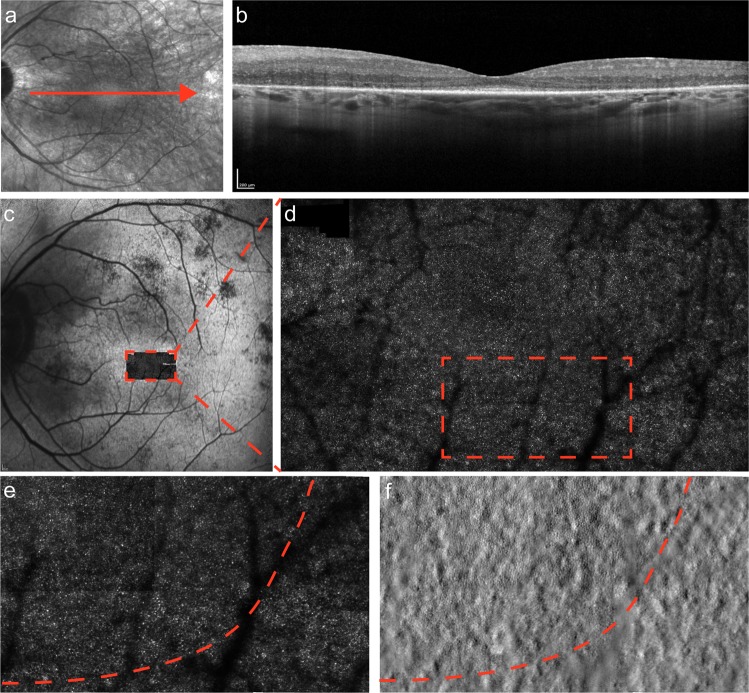
Multimodal imaging of *MYO7A*-associated type I Usher syndrome. **(a)** IR fundus photograph, with the central red arrow representing the OCT section shown in (**b**). **(c**) FAF photograph with confocal AOSLO imaging of the fovea and its surrounding region superimposed and magnified in (**d**). Further magnification of a peripheral area (dashed rectangle) in the confocal image (**e**), and its corresponding AOSLO split-detection image (**f**), shows the demarcation line between a contiguous mosaic of intact photoreceptors and their degenerating neighbours (dashed arc). It is important to note the stark contrast above and below this arc on the split-detection image (**f**), compared to the lack of difference in the confocal image (**e**)

AO-based imaging allows clinical diagnosis from early pathological changes and disease monitoring using the aforementioned metrics, thus conferring advantages that imaging systems prior to it had been unable to offer. The use of AO to study abnormalities in IRDs in conjunction with established imaging methods has enabled the cellular characterisation of these conditions (Figs. 5–7). We have collated the phenotypic findings for each IRD investigated thus far and categorised them based on their monogenetic disease-causing gene (Table 1). This provides a comprehensive and up-to-date summary of AO imaging findings in the IRDs.

**Table 1:** Summary of cellular findings using adaptive optics (AO) in inherited retinal diseases (IRDs) according to the disease-associated gene. Phenotypic characterisation studies applying AO in genetically-defined IRD patients were included following a literature search in February 2018. Diseases are listed in alphabetical order with nomenclature as per the online Mendelian inheritance in man (OMIM) database. Abbreviations: AO-FIO, adaptive optics flood illumination ophthalmoscopy; AOSLO, adaptive optics scanning laser ophthalmoscopy; RPE, retinal pigment epithelium; SD-OCT, spectral domain optical coherence tomography

Inherited Retinal Disease	Gene Symbol	Gene OMIM #	Phenotype OMIM #	Imaging Modality	Retinal Findings	Citations
Achromatopsia 2	*CNGA3*	#600053	#216900	AOSLO	Varied findings, from reduced cone density and reflectivity, to complete cone and rod mosaic.	[124]
Achromatopsia 3	*CNGB3*	#605080	#262300	AO-FIO AOSLO	Severely disrupted foveal and parafoveal photoreceptor mosaic. Residual foveal cone structure varies from sparse arrangement to absent. Presence of an ‘intraretinal bubble' noted in some patients.	[51, 73, 125]
Achromatopsia 4	*GNAT2*	#139340	#613856	AO-FIO AOSLO	Clearly defined parafoveal mosaic with slightly reduced foveal cone density. Residual cone density and reflectivity greater than in patients with CNGA3/CNGB3-associated achromatopsia.	[83, 126]
Bietti crystalline corneoretinal dystrophy	*CYP4V2*	#608614	#210370	AO-FIO AOSLO	Decreased cone density at parafoveal regions corresponding to RPE loss, with otherwise normal cone spacing. Microscopic bright spots visible, which correspond to small yellow-white crystalline deposits seen on fundoscopy; these are possibly residual cones over the crystals.	[127–129]
Blue cone monochromacy	*OPN1LW* *OPN1MW*	#300822 #300821	#303700	AOSLO	Decreased cone density, partly attributable to cone misidentification in areas with low cone reflectivity. On imaging with split-detection AOSLO, these areas contain remnant inner segment structures. In patients with L/M interchange mutations, a large foveal lesion was present with slightly improved parafoveal cone density as compared to those with a p.Cys203Arg mutation.	[130–135]
Bornholm eye disease	*BED*	#300843	#300843	AO-FIO AOSLO	Significant but variable degree of mosaic disruption and macular thinning. Cone density ranged from normal to reduced by over 75%. Two siblings showed different degrees of retinal thickness and cone mosaic disruption despite having the same disease-causing sequence variant.	[135, 136]
Bradyopsia	*RGS9* *R9AP*	#604067 #607814	#608415	AOSLO	Intact and structurally normal photoreceptor mosaic, in contrast with the clinically very similar oligocone trichromacy. In the patient with RGS9-associated disease, a hyporeflective lesion was seen at the foveal centre corresponding to an outer retinal defect, but with a normal mosaic elsewhere.	[6]
Central areolar choroidal dystrophy 2	*PRPH2/* *RDS*	#179605	#613105	AOSLO	Decreased foveal cone density with increased foveal reflectivity of the photoreceptor outer segment-RPE junction. Irregular cone packing and increased spacing. Reduced parafoveal cone density even in early stages of disease.	[137–140]
Choroideremia	*CHM*	#300390	#303100	AO-FIO AOSLO	Central island of relatively regular cone mosaic but with reduced density. Patches of RPE atrophy peripherally over which there is either abrupt loss of cones or remnant cone inner segments in outer retinal tubulations. Reduced peripheral photoreceptors with foveal sparing present in asymptomatic female carriers. Patchy cone loss seen in symptomatic female carriers.	[56, 57, 141–145]
Cone dystrophy with supernormal rod responses	*KCNV2*	#607604	#610356	AOSLO	Significantly reduced cone density, with the macula affected early in disease course. Groups of waveguiding cones are surrounded by patches of hyporeflectivity, representing absent or non-waveguiding cones.	[146]
Cone-rod dystrophy	*DFNB31*	#607084	-	AO-FIO	Mild decrease in parafoveal cone density compared to peripheral cone density.	[147]
Cone-rod dystrophy 14	*GUCA1A*	#600364	#602093	AOSLO	Range of findings, from near-normal cone density to widespread cone loss in younger patients. Few identifiable cones in older patients. An identical disease-causing sequence variant in a family (p.Ile143delinsAsnThr) can lead to different degrees of photoreceptor mosaic disruption, albeit with the same clinical features.	[148, 149]
Cone-rod dystrophy 20	*POC1B*	#614784	#615973	AO-FIO	Sparse cone density around central fovea with minimal detection of cones elsewhere. Hyperreflectivity in foveal areas containing residual cones.	[150]
Congenital stationary night blindness 1B	*GRM6*	#604096	#257270	AOSLO	Normal photoreceptor mosaic and normal density at all locations. Decreased parafoveal thickness of the inner retinal layers.	[104]
Doyne honeycomb retinal dystrophy	*EFEMP1*	#601548	#126600	AOSLO	Normal photoreceptor mosaic with hyperreflectivity in areas that correlate with drusen deposition.	[151]
Enhanced S-cone syndrome	*NR2E3*	#604485	#268100	AOSLO	Disrupted cone mosaic with varying reflectivity from each cone in the central retina. Increased cone spacing and larger cone size. Dark, patchy lesions seen in the macula.	[152, 153]
Fundus albipunctatus	*RDH5*	#601617	#136880	AOSLO	Decreased macular cone density with dark patches representing areas of cone loss that were not detected on fundoscopy or SD-OCT. Hyperreflective regions are surrounded by hyporeflective rings in areas corresponding to white dots seen on fundoscopy, which contain no photoreceptors or RPE cells.	[154–157]
Gyrate atrophy	*OAT*	#613349	#258870	AOSLO	Centrally preserved RPE and photoreceptors with normal cone density, with a peripheral ring of degenerated retina where no cones are seen. Slightly reduced cone density at the border between preserved and degenerated retina.	[158]
Late-onset retinal degeneration	*C1QTNF5*	#608752	#605670	AOSLO	Reticular pseudodrusen are seen as hyperreflective cores encapsulated by hyporeflective annuli, which underlie cone photoreceptors. Cones overlying lesions have widened inner segment diameters compared to those elsewhere.	[55]
Leber congenital amaurosis 1	*GUCY2D*	#600179	#204000	AOSLO	Decreased parafoveal cone density with sporadic hyporeflective cones. Enlarged residual cone inner segments seen using split-detection AOSLO.	[54]
Occult macular dystrophy	*RP1L1*	#608581	#613587	AO-FIO	Severe reduction in cone density and increased cone spacing seen in a ring-shaped distribution around the fovea. However, no cone abnormalities seen in asymptomatic family members with the same genetic variant (p.Arg45Trp).	[80, 159–163]
Oguchi disease 2	*GRK1*	#180381	#613411	AOSLO	Normal rod and cone mosaic, with normal foveal cone density. Increased perifoveal rod reflectivity during the light-adapted state compared to the dark-adapted state. Normal cone reflectivity.	[104]
Oligocone trichromacy	Unknown	-	-	AO-FIO	Irregular cone mosaic with reduced cone density peripherally, the extent of which is variable. Foveal cone outer segment length significantly decreased and peripheral retinal layer thinning seen. Normal-appearing cone mosaic seen in some patients with an oligocone trichromacy-like clinical phenotype.	[164–166]
Retinitis pigmentosa 4	*RHO*	#180380	#613731	AOSLO	Regions of increased cone spacing with a parafoveal annulus where cone identification is ambiguous. Frequent dark, patchy regions observed in mosaic where cones are mis-shapen.	[78, 167]
Retinitis pigmentosa 11	*PRPF31*	#606419	#600138	AOSLO	Intact central macular cone mosaic with normal cone density.	[168]
Retinitis pigmentosa 28	*FAM161A*	#613596	#606068	AOSLO	Severe diffuse cone atrophy with hyporeflective areas signifying sparse residual cones.	[169, 170]
Retinitis pigmentosa 62	*MAK*	#154235	#614181	AOSLO	Normal cone spacing in early disease, and increased cone spacing in advanced disease with maintenance of central foveal cone structure until late stages.	[171]
Retinitis pigmentosa 65	*CDHR1*	#609502	#613660	AOSLO	Increased cone spacing and reduced cone density in focal parafoveal regions.	[172]
Retinitis punctata albescens	*RLBP1*	#180090	#136880	AO-FIO	Disorganised cone mosaic with increased cone spacing and empty spaces. Below these spaces, parafoveal white dot-like deposits are seen as elongated structures, which are not present in the fovea.	[173]
Stargardt disease 1	*ABCA4*	#601691	#248200	AOSLO	Variable foveal cone mosaic. Central atrophy with annulus of hyporeflectivity present in some patients, whereas others have a contiguous foveal mosaic with enlarged cone size and reduced density. Hyperreflective areas present that do not correlate with lipofuscin deposition seen on auto-fluorescence. Outer retinal tubulations containing photoreceptor-like structures are seen.	[4, 53, 103, 174–178]
Stargardt disease 3	*ELOVL4*	#605512	#600110	AO-FIO	Diffuse loss of central cone reflectivity with decreased density in early disease. Bull’s eye pattern of parafoveal decreased cone density seen in advanced disease.	[179]
Tritan colourblindness	*OPN1SW*	#613522	#190900	AO-FIO	Variability in cone mosaic, with near-normal S-cone density in early life progressing to complete S-cone loss in later life. Cone density remains in normal range due to low proportion of S-cones.	[180]
Usher syndrome 2A	*USH2A*	#608400	#276901	AOSLO	Reduced foveal cone density. Foveal clusters of low reflectivity cones seen with diseased outer segments. Remnant inner segments seen within dark patches using split-detection AOSLO.	[23]
Usher syndrome 3A	*CLRN1*	#606397	#276902	AOSLO	Preserved central foveal cone density, which abruptly ends to peripheral photoreceptor degeneration. In advanced disease, RPE cells seen in areas where the cone mosaic is absent or outer segments are abnormal.	[181]
Vitelliform macular dystrophy 2	*BEST1*	#607854	#153700	AOSLO	Highly variable depending on disease stage, with hyporeflective patches of mosaic disruption correlating with the vitelliform lesion location. Split-detection AOSLO shows remnant inner segments of photoreceptors in these areas with varied morphology. Well-preserved photoreceptor density present in areas adjacent to lesions and elsewhere, except where subretinal fluid is present.	[182–185]
X-linked atrophic macular degeneration	*RPGR*	#312610	#300834	AOSLO	Variable cone mosaic, from normal appearance to asymmetrical shapes and irregularity. Small areas of varying rod loss and rod hyperreflectivity seen in patients, either with normal-appearing or reduced density mosaics. RPE-like cells seen at the boundaries of intact mosaic.	[66, 68, 186, 187]
X-linked retinoschisis	*RS1*	#300839	#312700	AO-FIO AOSLO	Canal-like foveal schisis cavities seen, with a spoke wheel pattern of inner retinal folds. Increased parafoveal and cavity cone spacing. Normal photoreceptor mosaic elsewhere up until the edges of cavities. Widened cone diameters noted in macula due to inner segment swelling.	[188–192]

## Limitations and future prospects

The heterogeneity and rare prevalence of IRDs necessitates larger cohort sizes in AO-based clinical studies, for which multicentre collaborations are required [118]. However, inter-centre discrepancies, in the form of differing AO-aided imaging modalities and image acquisition methods, complicate collaborative efforts. Aside from difficulties in accurate cone identification, the impracticality of manual image grading by observers and its poor repeatability can also render metric applications as unreliable [95]. Recent efforts to produce automated analytic tools for AO images have shown promise in both confocal and non-confocal settings [119, 120], with the latter being used in achromatopsia and Stargardt disease [121, 122]. Although further work is needed to characterise the performance of these algorithms in relation to different metrics [123], this provides AO retinal imaging with good prospects in fulfilling their clinical potential.

In summary, the correction of optical aberrations using adaptive optics has enabled high-resolution microscopic visualisation of the living retina, thereby furthering our understanding of IRD pathogenesis. Despite drawbacks in cost and technical complexity, the benefits of applying AO in this context are vast, including early diagnosis, detection of subclinical disease changes, patient stratification for treatment and assessment of treatment efficacy. The challenge for the coming years is in transferring this tool from the research laboratory to the clinical setting, where it could transform patient outcomes in both the IRDs and other retinal diseases.

## Method of literature search

A literature search was conducted in February 2018 using the term ‘adaptive optics’ on Pubmed in combination with each of the following key terms: ‘retinal imaging’, ‘retina’, ‘cones’ and ‘photoreceptors’. This generated 687 publication results, of which 79 were directly related to IRDs. For each IRD, the condition name (e.g. Stargardt disease) was additionally searched in conjunction with ‘adaptive optics’ on Pubmed. Finally, abstracts presented at previous Association for Research in Vision and Ophthalmology (ARVO) annual meetings were reviewed using the search term ‘adaptive optics’. This yielded 1,602 results, of which 81 were directly related to IRDs.
